# Mainland and island populations of *Mussaenda kwangtungensis* differ in their phyllosphere fungal community composition and network structure

**DOI:** 10.1038/s41598-020-57622-6

**Published:** 2020-01-22

**Authors:** Xin Qian, Shengchun Li, Binwei Wu, Yonglong Wang, Niuniu Ji, Hui Yao, Hongyue Cai, Miaomiao Shi, Dianxiang Zhang

**Affiliations:** 1 College of Life Sciences, Fujian Agriculture and Forestry University, Fuzhou, 350002 China; 2 Key Laboratory of Plant Resources Conservation and Sustainable Utilization, South China Botanical Garden, Chinese Academy of Sciences, Guangzhou, 510650 China; 3 University of Chinese Academy of Sciences, Beijing, 100049 China; 4 Center of Conservation Biology, Core Botanical Gardens, Chinese Academy of Sciences, Guangzhou, 510650 China

**Keywords:** Microbial ecology, Fungal ecology

## Abstract

We compared community composition and co-occurrence patterns of phyllosphere fungi between island and mainland populations within a single plant species (*Mussaenda kwangtungensis*) using high-throughput sequencing technology. We then used 11 microsatellite loci for host genotyping. The island populations differed significantly from their mainland counterparts in phyllosphere fungal community structure. Topological features of co-occurrence network showed geographic patterns wherein fungal assemblages were less complex, but more modular in island regions than mainland ones. Moreover, fungal interactions and community composition were strongly influenced by the genetic differentiation of host plants. This study may advance our understanding of assembly principles and ecological interactions of phyllosphere fungal communities, as well as improve our ability to optimize fungal utilization for the benefit of people.

## Introduction

The phyllosphere provides a unique habitat for a diverse community of microorganisms that live within or on the surface of leaves^1^. Phyllosphere fungi have been considered pivotal determinants of host-plant fitness, productivity, and ecosystem functioning^2,3^. For example, phyllosphere fungi may confer salt, heat or herbivory tolerance to host plants^4–6^, they may potentially be a source of plant diseases due to the pathogenic phases in their life cycles^7,8^, or they may act as initial decomposers of leaf litter and play important roles in nutrient cycling^9,10^. With advances in high-throughput sequencing technologies where DNA samples can be extracted directly from leaves, phyllosphere fungal communities have been examined to determine the community composition and potential drivers of this high diversity^11^. Yang *et al*.^12^ found that leaf carbon was the main driver of changes for the foliar fungal community composition of *Betula ermanii* along a subalpine timberline. Zimmerman *et al*.^13^ showed that climate factors such as temperature and rainfall strongly structured fungal communities in the leaves of *Metrosideros polymorpha* across a Hawaiian landscape. A different study conducted in tropical lowland rainforests focused on the factors shaping the community structure of phyllosphere fungi and found that plant traits and taxonomy were critical factors^14^. Moreover, several studies have explored the relationship between the genetic identity of a conspecific host and the community composition of its phyllosphere fungi, but no consensus was established. Variation in phyllosphere fungal community composition in *Fagus sylvatica* and *Mussaenda pubescens* var. *alba* was mostly explained by host genotype^15,16^. In contrast, plant genotype had no significant effect on the phyllosphere fungal microbiome of *Picea glauca*^17^.

Microorganisms often vary across trophic modes and functionally distinct niches, which allows them to co-exist and to form complex ecological networks comprising microbial members that interact with each other^18,19^. A comprehensive exploration of co-occurrence networks is critical to understanding the assembly process and function of the microbial community, because it provides insights into potential relationships and reveals niche spaces. Further, exploration may uncover possible keystone taxa that exert a considerable impact on community structure and function, regardless of their abundance^20,21^. Co-occurrence network analysis based on high-throughput sequencing data has been increasingly applied to examine the ecological interactions among microorganisms in different habitats including water environments^22–24^, human and animal digestive tracts^25,26^, soil^27,28^, and tree trunks^29^. Nevertheless, compared to bacterial communities, far fewer studies have been conducted to investigate co-occurrence patterns of fungal microbiome especially in plant associated communities. Recently, Bouffaud *et al*.^30^ demonstrated that across the European regions, closely-related arbuscular mycorrhizal fungi (AMF) tended to co-occur with a significantly higher probability than distantly-related ones. Qian *et al*.^31^ found a trend of reduced connectivity in phyllosphere fungal co-occurrence networks with increasing elevation. However, we still lack fundamental knowledge concerning factors that are predictive of differences among network structures.

Allopatric isolation on islands has long been considered a driver of evolutionary diversification^32,33^. Moreover, groups of islands may act conveniently as replicates in which general evolutionary patterns can be distinguished from unique outcomes^34^. Many studies have presented the idea that island populations are inclined to display a significant genetic separation from corresponding species on the mainland due to their small population size, founder effects and limited immigration^35^. For instance, geographical isolation by sea may have effectively led to genetic differentiation in *Weigela coraeensis* between the Honshu mainland and the Izu islands, although gene flow was still occurring between the mainland and the islands^33^. Remarkable genetic differentiation was also observed in *Periploca laevigata*, albeit the overall genetic diversity in plant taxa did not differ significantly between mainland and island populations^36^. In addition, the degree of genetic divergence also depends on when plants emigrated to the islands, on the distance from island to mainland, and on biological traits, including seed dispersal ability and reproductive systems^33^. Islands can also be natural laboratories for studying community assembly and the processes that shape species distributions and interactions^37^. However, most island biogeography studies have focused on macro-organisms, so information about the distribution pattern of plant associated fungal microbiomes is quite limited. Recently, Davison *et al*.^38^ found that island AMF communities consist of few endemic taxa, but are as diverse as mainland ones. This indicates that efficient dispersal outweighs potential negative effects from taxogenesis and extinction on islands. Whereas da Silva *et al*.^39^ showed that AMF community assemblages differed significantly between island and mainland environments due to climatic differences, as well as edaphic and spatial factors. Nonetheless, the island and mainland patterns of phyllosphere fungal distribution have seldom been studied, and thus the relationships between their community structure and host plant genetic differentiation are poorly characterized.

*Mussaenda kwangtungensis* H. L. Li (Rubiaceae) is a drought-resistant shrub, 1–2.5 m high, distributed across mainland and islands in Guangdong Province, China; This plant and this location present an ideal study system to compare distribution patterns of phyllosphere fungal communities and host genetic differentiation between island and mainland populations. *M. kwangtungensis* has historically been used in Chinese traditional herbal medicine as an antichloristic and antipyretic agent against laryngopharyngitis, acute gastroenteritis and dysentery^40^. Our prior efforts have investigated its reproductive characteristics as well as its population genetics^40,41^. However, little is known about the distribution pattern of phyllosphere fungal microbiome associated with this plant species. In this study, we used high-throughput sequencing of fungal ribosomal internal transcribed spacer 2 (ITS2) on the Illumina MiSeq platform to analyze the phyllosphere fungal community structure and the co-occurrence network characteristics of island and mainland *M. kwangtungensis* populations. Then, we also examined the host population genetic structure and plant genotyping using 11 nuclear DNA microsatellite loci. The main aims of this study were to: (1) investigate whether the community composition of the island phyllosphere fungi dffered significantly from their mainland counterparts; (2) explore the co-occurrence patterns of phyllosphere fungal microbiomes in island and mainland populations, respectively; (3) evaluate the importance of host genetic structure on variation in the fungal community composition and co-occurrence network structure of a single plant species with an island-mainland distribution.

## Material and Methods

### Study site and sampling

**Figure 1:**
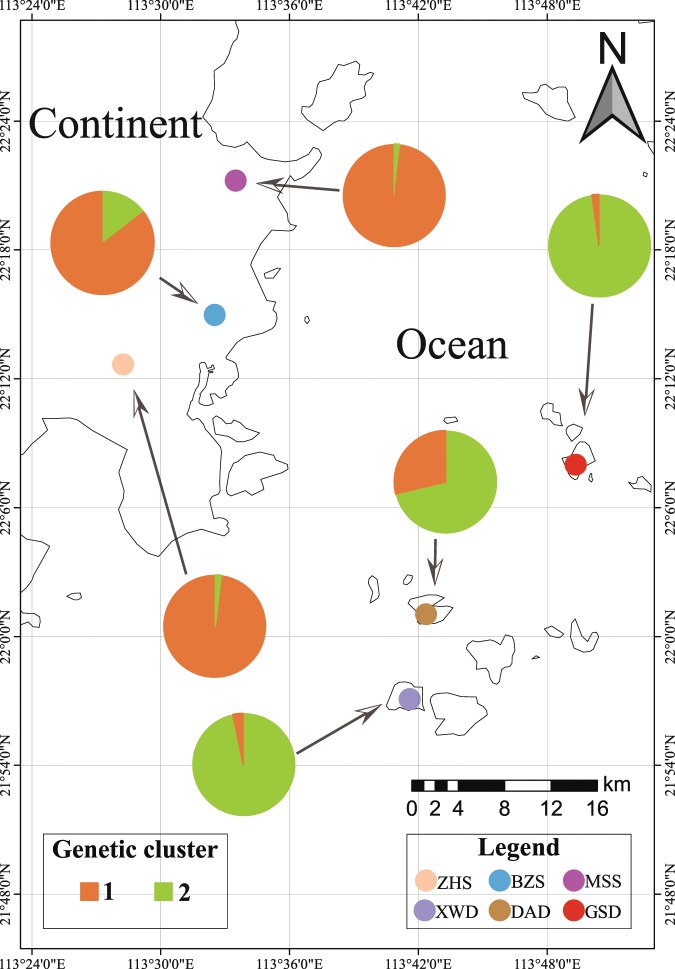
Map of the study region and the results of genetic clustering based on DNA microsatellite data.

This study was conducted at three island and three mainland sites having *Mussaenda kwangtungensis* populations in Guangdong Province, South China (Fig. 1, Table S1). At each sampling site, we recorded the geographic coordinates (latitude and longitude) and elevation with a handheld GPS unit. Mean annual temperature (MAT) and mean annual precipitation (MAP) of each population were obtained from the WorldClim global climate data set with a high spatial resolution of 30 seconds (http://worldclim.org/version2).

Ten mature and healthy individuals (>20 m away from each other) were selected randomly for sampling at each site during anthesis, which brought the total of 60 individuals across all sites. With scissors and while wearing sterilized gloves, we collected nine asymptomatic sun leaves from three current-year shoots growing in different directions from each plant. Samples were then packed into sterile polyethylene bags containing sufficient silica gel (>300 g) for quick drying.

### DNA library preparation and MiSeq sequencing

Leaves were pooled at the individual plant level and were then pulverized mixed in liquid nitrogen using sterilized pestles and mortars under aseptic conditions in a laminar airflow to avoid external contamination. We extracted DNA from approximately 500 mg of leaf powder using the PowerSoil DNA Isolation kit (MoBio Laboratories, CA, USA) according to the manufacturer’s instructions. DNA quality and quantity were measured on a NanoDrop 1000 spectrophotometer (Thermo Scientific, Wilmington, USA).

A two-step PCR procedure was used to prepare amplicon libraries for the Illumina Miseq sequencing platform. First, the entire fungal internal transcribed spacer (ITS) region was amplified using the conventional primers ITS1F^42^ and ITS4^43^. PCR amplification was performed in a 25 μL reaction with 0.5 μM of each primer, 2.5 µL Phusion HF Buffer, c. 10 ng DNA template, 250 μM of dNTP Mix, and 1 Unit Phusion DNA polymerase (New England Biolabs, Hitchin, UK). The PCR thermal profile was as follows: initial denaturation at 94 °C for 5 min, 20 cycles of 94 °C for 1 min, 56 °C for 50 s and 68 °C for 30 s, and a final extension at 68 °C for 10 min. The PCR products were then diluted 50 times with sterile deionized water, and 1.0 μL of the resulting solution was then used as a template for the second PCR round to ITS2 region, using the same conditions except for primers fITS7^44^ and ITS4 linked with 12 base pair (bp) barcode sequences. Thermocycling profile was also identical to those used in the first PCR round. To minimize PCR biases, each sample was amplified in triplicate and the three replicate products were grouped together into a general sample. The entire PCR product was separated on a 1.5% agarose gel and the target fragment was excised and purified using a MinElute PCR Purification Kit (Qiagen, Venlo, the Netherlands). Then, the DNA samples were pooled and concentrated using the DNA Clean and Concentrator kit (Zymo Research, Orange, CA, USA) and adjusted to 10 ng μL^−1^. The sequencing library was constructed with the Illumina sequencing adaptor using the Illumina TruSeq DNA PCR-Free LT Library Prep Kit (Illumina, CA, USA) according to the manufacturer’s instructions. The library was applied to an Illumina MiSeq PE250 platform for sequencing using the paired end (2 × 250 bp) option, and was conducted at the Environmental Genomic Platform of Chengdu Institute of Biology, Chinese Academy of Sciences, China. The sequences have been submitted to the NCBI Sequence Read Archive (SRA) with accession number SRP226848 and PRJNA579231.

### Microsatellite genotyping

The genomic DNA was also used for host plant genetic analysis. Microsatellite loci have been developed by Duan *et al*.^45^. Eleven loci showed polymorphisms in *M. kwangtungensis* populations (Table S2). Each forward primer was fluorescently labelled with TAMRA, FAM, HEX, or ROX (Invitrogen, CA, USA) for further screening. The PCR was carried out in a 10 μl reaction solution including 35 ng of genomic DNA, 3.5 μL deionized water, 0.2 μM of each primer pair and 5 μL PCR reaction Mix (Tiangen, Guangzhou, China). The cycling conditions for the amplification were set at 94 °C for 5 min, then 35 cycles of denaturation at 94 °C for 30 s, 30 s at annealing temperature (the value of each primer is shown in Table S2), and then extension at 72 °C for 1 min, with a final extension of 8 min at 72 °C. In addition, a touchdown PCR program was used for four primers (AC30, CT113, CT135, and CT142) with initial denaturation for 5 min at 94 °C, 7 touchdown cycles of 94 °C for 30 s, 30 s at 60 °C with decreasing annealing temperatures in decrements of 1 °C per cycle and 72 °C for 60 s, and then 28 cycles of 94 °C for 30 s, 53 °C for 30 s, and 72 °C for 60 s. The final extension was done at 72 °C for 8 min. PCR products were separated by an ABI PRISM 3100 Genetic Analyser (Applied Biosystems, CA, USA). Genotyping data was obtained from allele binning and calling using GeneMarker ver. 2.4.0 (SoftGenetics LLC, Pennsylvania, USA).

### Host population genetic structure

Observed heterozygosity (*Ho*) and hierarchical analysis of molecular variance (AMOVA) were estimated to quantify the genetic diversity and assess genetic differentiation among populations from the two geographical regions (the mainland and the islands) using Arlequin ver. 3.5^46^. Nei genetic distance^47,48^, and principal coordinates analysis (PCoA) were determined with the GenAlEx 6.502^49^. Nei genetic distance was then employed to construct a UPGMA dendrogram to visualize the genetic relationships among the populations. Isolation-by-distance (IBD) effects were tested based on pairwise genetic and geographic distances at the population level by using the “ecodist” R package^50^. Population structure was explored using the program STRUCTURE 2.3.4^51^. Ten replicates of an admixture model assuming genetic groups ranging from 1 to 6 with admixture and dependent allele frequencies were performed with 100 000 Markov Chain Monte Carlo (MCMC) burn-in steps followed by 50 000 iterations. Program STRUCTURE HARVESTER^52^ was then used to determine the optimal number of clusters (K). The results of the replicates at the best number of clusters were combined and analyzed with the Full Search algorithm using CLUMPP^53^. Finally, the corresponding Q matrices were visualized by DISTRUCT program^54^.

### Bioinformatics

The raw sequence data was filtered using PEAR^55^ and QIIME^56^ programs to merge paired-end sequences, to remove low quality sequences, and to demultiplex all sequences into each sample. The ITS2 region was detected and extracted using the fungal ITSX software package^57^, and de novo chimera detection and deletion were performed in the UCHIME algorithm^58^. The remaining sequences were then binned into operational taxonomic unites (OTUs) at a 97% sequence similarity level by employing the UPARSE pipeline^59^ after dereplication and discarding all singletons. A representative sequence (the most abundant) of each OTU was selected and searched against the UNITE database using a BLAST algorithm to determine the taxonomic identity of fungal OTUs^60^. Ten best-matching references were considered to taxonomically annotate OTUs as accurately as possible. The results of the BLASTn search were considered reliable for robustly assigning sequences to fungal Kingdom if e-values < e-50. Therefore those with > e-50 were eliminated. Sequence identities of 90%, 85%, 80% and 75% were used as criteria for assigning OTUs to the taxonomic levels of genus, family, order and class, respectively^60^. The OTUs belonging to mycorrhizal fungi or animal pathogens were excluded because they might be contaminated. The OTUs with less than 10 reads were then excluded in all the samples because their sequences might have contained PCR or sequencing errors^61^. To eliminate the effects of sequence number variation from the different samples prior to downstream analysis, the number of sequences was rarifed to the minimum sequencing depth of each sample using MOTHUR^62^ through a subset of randomly selected reads.

### Statistical analysis of fungal community composition

Most statistical analyses were conducted in R v.3.3.3^63^. Rarefaction curves for all phyllosphere fungi at island and mainland regions were computed to evaluate the comprehensiveness of the sampling strategy using the “vegan” R package^64^. After rarefying based on the smallest read number from any individual sample (5415 reads per sample), 324900 reads remained, representing 772 fungal OTUs in the phyllosphere of *M. kwangtungensis* populations. Because of the rejection of homoscedasticity by the Levene’s test for equal variance, the non-parametric two-sample Wilcoxon test^65^ was used to compare the OTU richness between mainland and island populations. Simple linear regression analysis was conducted to test the relationship between fungal OTU richness and plant genetic diversity. The distance matrix for the phyllosphere fungal community composition (Hellinger-transformation of the OTU read data) was constructed by calculating dissimilarity with the Bray-Curtis method^66^. The Bray-Curtis matrix was used to perform hierarchical clustering analysis of fungal communities across different plant populations. To investigate patterns of phyllosphere fungal community structure, nonmetric multidimensional scaling (NMDS) ordination of analysis was performed based on Bray-Curtis dissimilarity. Then, differences between the mainland and island fungal community compositions were tested by conducting analysis of similarity (ANOSIM) and a permutational multivariate analysis of variance (PERMANOVA). Furthermore, Linear Discriminant Analysis (LDA) effect size (LEfSe, http://huttenhower.sph.harvard.edu/galaxy/)^67^ was employed to determine indicator OTUs for each of the two regions. A logarithmic LDA score of 3.0 was set as the threshold for discriminative features.

Principal coordinate analysis was used to convert the pairwise Nei genetic distances to plant genetic eigenvectors (PGE) by using the “vegan” R package. The spatial principal coordinate of neighbor matrices (PCNM) vectors with positive eigenvalues were obtained based on the transformation of geographic distance (latitude and longitude) via the “PCNM” R package^68^. Significant spatial PCNM and host PGE vectors were forward-selected (α = 0.05) using the “Packfor” R package^69^ prior to subsequent statistical analyses. A PERMANOVA^70^ performed on the community matrix (Bray-Curtis dissimilarities) was used to evaluate the effect of host genetic eigenvectors, geographic distance, and climate factors on phyllosphere fungal community structure, which is also implemented through the “vegan” package with population site as a random effect. Moreover, multispecies generalized linear models (GLM) fitted with the “mvabund” R package were also used to identify the main drivers of phyllosphere fungal community structure, as Bálint *et al*.^71^ did. Congruence among distance matrices (CADM)^72^ and the Mantel test were then used to identify congruent patterns between host genetic structure and fungal community structure in mainland and island populations.

### Fungal co-occurrence networks

Co-occurrence analyses were implemented to obtain a better understanding of fungal interactions in the phyllosphere of *M. kwangtungensis*. The fungal networks were built up with the “WGCNA” R package^73^ based on the Spearman correlation index. The nodes and the edges in the network represent fungal OTUs and the significant interactions between pairs of OTUs, respectively. The OTUs with relative abundances less than 0.01% were filtered because they were poorly represented. The *P*-values for multiple testing were calculated using the Benjamini and Hochberg discovery rate (FDR) test controlling procedure^74^. Only the rank correlation coefficient with values above 0.6 or below −0.6 and a statistically significant *P* value lower than 0.05 were considered as a valid correlation in the network. Sub-networks for mainland and island habitats, and then for each individual sample from the meta-community network, were then identified by preserving fungal OTUs present in each plant using the “igraph” package^75^. The networks of the two habitats were graphically displayed in Gephi (http://gephi.github.io/). Erdös-Réyni model random networks^76^ with the same number of nodes and edges as the observed networks were also constructed for each habitat. Network topological parameters (number of nodes and edges, average degree, degree centralization, centralization closeness, and modularity) provided in the “igraph” R package were calculated for each sample sub-network. The Wilcoxon test was then employed to assess significant difference in measured topological parameters between island and mainland networks^77^. The importance of biotic and abiotic factors (host plant genetic eigenvectors, geographic distance, and climate factors) for network-level topological features was evaluated with a random forest analysis using the “randomForest”^78^ and “rfPermute”^79^ R packages. The connectivity of network node was determined by its value of within-module connectivity (*Zi*) and among-module connectivity (*Pi*), which were calculated based on the methods of metabolic networks^80^. Node topologies were classified into four categories, according to the definition by Poudel *et al*.^81^: peripherals (*Zi* < 2.5 and *Pi* < 0.62, nodes with few links to other species), connectors (*Pi* > 0.62, nodes that connect modules), module hubs (*Zi* > 2.5, highly connected nodes within modules,), and network hubs (*Zi* > 2.5 and *Pi* > 0.62, highly connected nodes both in general and within a module). Connectors, module hubs, and network hubs were called keystone OTUs, which were considered to play unique and crucial roles in the stability of the fungal co-occurrence network structure and functioning^20,82^.

### Compliance with ethical standards

This article does not contain any studies with human participants or animals performed by any of the authors.

## Results

### Fungal taxon identification

**Figure 2:**
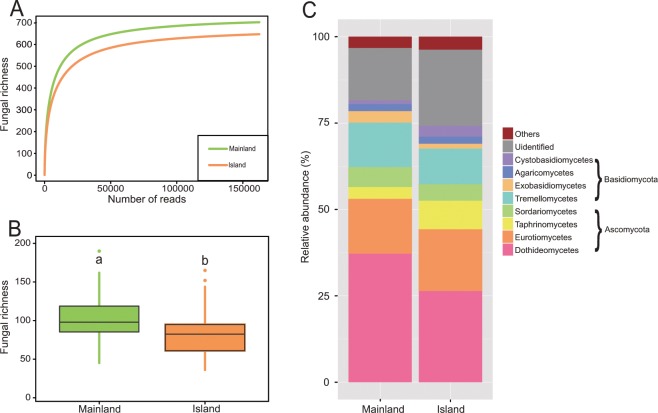
(**A**) Rarefaction curves and (**B**) box plots of phyllosphere fungal OTU richness in mainland and island *Mussaenda kwangtungensis* populations. Each point represents an individual plant. Different letters indicate statistical significance at *P* < 0.05. (C) Taxonomic composition of the fungal communities at the class level. The taxa with relative abundances of <1% were grouped into “others”.

The rarefaction curves of the observed fungal OTUs reached a saturation plateau in both island and mainland populations, indicating that the majority of the phyllosphere fungal diversity was sampled (Fig. 2A). An average of 94 ± 4 OTUs (mean ± SE) per individual plant was found with 101 ± 6 OTUs per mainland sample and 86 ± 6 OTUs per island sample. The result of the Two-Sample Wilcoxon test showed that fungal richness was significantly lower on islands than the mainland (Fig. 2B). Fungal richness demonstrated a significantly positive correlation with plant genetic diversity (R^2^ = 0.645, *P* = 0.033). The observed OTUs were mainly found to belong to two fungal phyla: Ascomycota (529 OTUs; 61.3% of the reads) and Basidiomycota (199 OTUs; 21.1%). At the class level, the Ascomycota fungal reads recovered were dominated by the Dothideomycete (279 OTUs; 21.1%) and Eurotiomycetes (61 OTUs; 16.8%), while the most common Basidiomycota class was Tremellomycetes (60 OTUs; 11.6%) (Fig. 2C). In total, 260 genera were identified in phyllosphere fungal community of *M. kwangtungensis*. The most abundant genera (Top 5) were *Aureobasidium* (5 OTUs; 5.6%), *Phaeococcus* (1 OTU; 4.4%), *Cladosporium* (2 OTUs; 2.9%), *Phaeophleospora* (5 OTUs; 2.7%)and *Phyllosticta* (6 OTUs; 2.4%).

### Population structure of host plant

**Figure 3:**
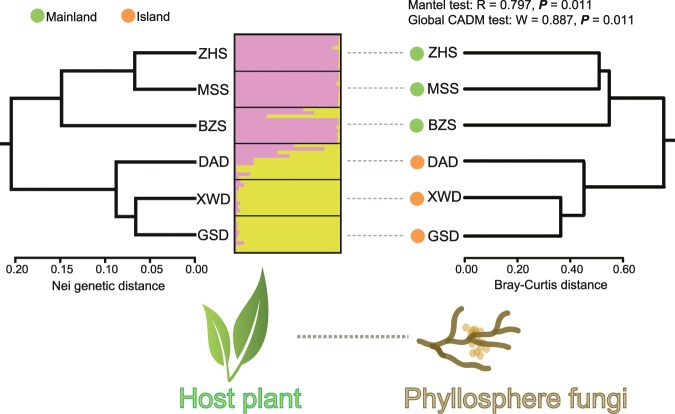
Topological congruencies between host genetic cluster and fungal dendrogram relationships. Left panel: a UPGMA dendrogram of host populations based on Nei genetic distance. Bar plot: population genetic structure of host plants using STRUCTURE analysis. Each thin vertical bar represents an individual plant, showing the proportion of its genetic pool assigned to each cluster. Right panel: a hierarchical dendrogram tree of the phyllosphere fungal communities based on Bray–Curtis dissimilarity.

The eleven microsatellite loci showed an overall *Ho* of 0.609 for mainland populations of *M. kwangtungensis*, and 0.516 for island region. The AMOVA results demonstrated significant genetic differentiation between mainland and island populations (F_CT_ = 0.099, *P* < 0.001). The IBD test revealed no significant correlation between population genetic and geographic distances (r = 0.529, *P* = 0.100). Delta K (ΔK) values from STRUCTURE analysis peaked sharply at K = 2, which indicates that there were two distinct genetic clusters, corresponding closely to the mainland and island regions (Fig. S1; Fig. 3). The UPGMA tree based on Nei genetic distance clustered all populations into two major groups (Fig. 3) with the mainland populations (BZS, ZHS and MSS) forming one group, and the remaining island populations (DAD, XWD and GSD) forming the other group. In accordance with the STRUCTURE and UPGMA cluster analysis, principal coordinate analysis also separated the populations into two main groups. The group coincided with the continental and insular habitats along Axis 1, and accounted for 73.2% of the total genetic variability (Fig. S2).

### Fungal community composition

**Figure 4:**
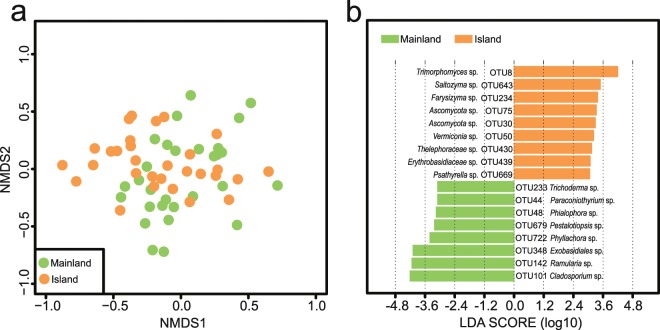
(**a**) Non-metric multidimensional scaling (NMDS) ordination of the fungal community structure. (**b**) Histogram of the linear discriminant analysis (LDA) scores computed for features differentially abundant between mainland and island populations with effect size measurements (LEfSe). Only OTUs with a LDA score greater than 3.0 are displayed.

The NMDS plot showed an observed structuring of phyllosphere fungal community between mainland and island populations based on Bray-Curtis dissimilarity (Fig. 4a). The significant differentiation was statistically confirmed by the results of ANOSIM (R = 0.100, *P* = 0.002) and PERMANOVA (R^2^ = 0.045, *P* = 0.002) analyses (Fig. 4a). The variation in fungal community structure was mainly explained by host plant genetic eigenvectors (14.2%) and the climate factor MAP (2.2%) (Table 1). These results closely corresponded to the multispecies GLM, where host genetic structure and MAP exerted the largest and second largest impacts on fungal community composition (Table 1). The global CADM test rejected the null model of incongruence between the matrices (*P* < 0.05), suggesting significant congruence between host population genetic structure and fungal dendrogram relationships (Fig. 3). The Mantel test results revealed a strong correlation between host genetic distance and phyllosphere fungal community dissimilarity (R = 0.797, *P* = 0.011; Fig. 3).

**Table 1:** Effects of host and abiotic variables on the community composition of phyllosphere fungi revealed by permutational multivariate analysis of variance (PERMANOVA) and multispecies generalized linear models (GLM).

Variable	PERMANOVA			Multispecies GLM	
	SS	R^2^	*P* value	TS	*P* value
Host genetic eigenvectors	2.383	0.142	<0.001***	4781	0.010**
Geographic distance	0.377	0.023	0.055	1851	0.535
MAP	0.366	0.022	0.006**	1655	0.040*
MAT	0.265	0.016	0.599	2468	0.188
Elevation	0.291	0.017	0.364	1335	0.980

SS: sum of squares; TS: test statistics; MAT: mean annual temperature; MAP: mean annual precipitation; *: 0.05 < *P* < 0.01; **: 0.01 < *P* < 0.001; ***: *P* < 0.001.

Seventeen fungal OTUs were found to respond significantly to the two habitat categories, with eight indicator OTUs (*Cladosporium* sp., *Ramularia* sp., *Exobasidiales* sp., *Phyllachora* sp., *Pestalotiopsis* sp., *Phialophora* sp., *Paraconiothyrium* sp., and *Trichoderma* sp.) for mainland and nine (*Trimorphomyces* sp., *Saitozyma* sp., *Farysizyma* sp., *Ascomycota* sp. (OTU 75), *Ascomycota* sp. (OTU 30), *Vermiconia* sp., *Thelephoraceae* sp., *Erythrobasidiaceae* sp., and *Psathyrella* sp.) for island (Fig. 4b).

### Fungal co-occurrence network

**Figure 5:**
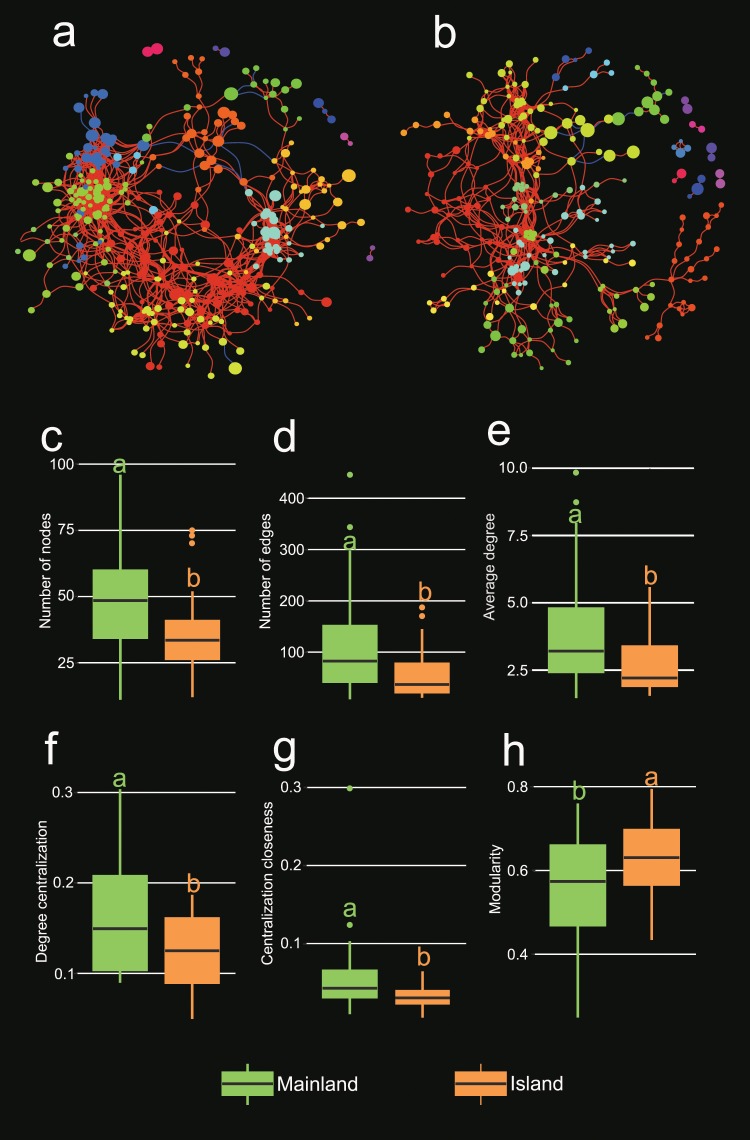
Co-occurrence networks of phyllosphere fungal communities in mainland (**a**) and island (**b**) populations. Each node represents a fungal OTU and is colored by module. The size of the node is proportional to the number of reads. The color of each link reflects positive (red) or negative (blue) associations. (**c**–**h**) Comparison of main network properties between the two different regions. Different letters indicate statistical significance at *P* < 0.05.

Both mainland and island networks displayed a scale-free degree distribution (Fig. S2), suggesting that most phyllosphere fungal OTUs had low-degree values, and only a few hub nodes had a large number of connections to other members. Each network showed overwhelmingly more positive associations (99.8% and 98.8%, in mainland and island network, respectively) than negative ones (Fig. 5a,b). Main network-level topological features of each individual subnetwork were calculated to compare the co-occurrence patterns between mainland and island regions. There were significantly more nodes and edges, and a greater average degree for mainland networks than island ones (Fig. 5c–e). This indicates that the island habitat has a less complex structure. Degree centralization, and centralization closeness of individual sub-networks were higher in the mainland than the island regions (Fig. 5f,g), suggesting that the phyllosphere fungal networks were more connected in the mainland than the island. In contrast, modularity values were higher in island networks than mainland ones (Fig. 5h). This indicates that the island networks had more fragmented and modular structures. The random forest modelling outcomes demonstrated that plant genetic eigenvectors (PEGs) were the most important contributors to all the involved network topological features (Table 2). Additionally, MAP was the second most important factor explaining the variation in number of nodes. Elevation and MAP had a significant impact on the number of edges and degree centralization.

**Table 2:** Mean predictor importance of host and abiotic variables on network structure of phyllosphere fungal community revealed by random forest analyses.

Network property	Variable	Importance	*P* value
Number of nodes	PGE21	7.039	0.029
	PGE28	4.648	0.049
	PGE1	4.077	0.029
	MAP	2.592	0.030
Number of edges	PGE28	5.834	0.004
	Elevation	4.197	0.002
	PGE31	3.731	0.042
	MAP	2.949	0.010
Average degree	PGE21	6.161	0.011
	PGE11	4.190	0.044
	PGE30	4.104	0.042
Centralization closeness	PGE25	3.277	0.039
	PGE22	3.068	0.049
Degree centralization	PGE2	8.968	0.002
	PGE33	5.748	0.012
	PGE31	4.994	0.026
	Elevation	4.790	0.006
	MAP	2.945	0.032
Modularity	PGE34	5.441	0.024
	PGE2	4.054	0.028

PGE: plant genetic eigenvector; MAP: mean annual precipitation. Only significant variables were shown.

**Figure 6:**
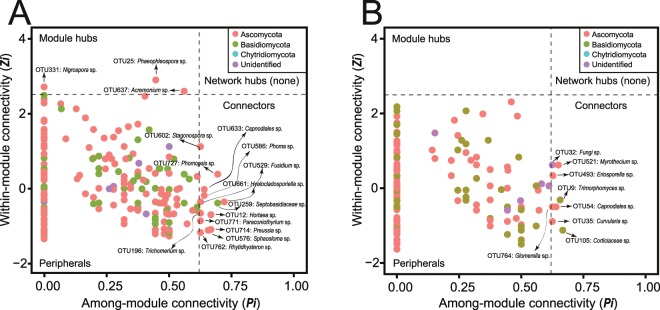
*Zi*-*Pi* plots to identify putative keystone OTUs within the phyllosphere networks in mainland (**A**) and island (**B**) plant populations. Each point represents a fungal OTU. Threshold values of *Zi* and *Pi* for classification are 2.5 and 0.62, respectively. The modules hubs and connectors are listed on the plots.

The majority of the nodes in both mainland (95.5%) and island (96.9%) networks were peripherals with most of their links inside their modules (Fig. 6). Neither mainland nor island networks possessed network hubs highly connected with other nodes within entire network (Fig. 6). Three module hubs (*Nigrospora* sp., *Phaeophleospora* sp., and *Acremonium* sp.) and 13 connectors (*Stagonospora* sp., *Capnodiales* sp., *Phomopsis* sp., *Phoma* sp., *Fusidium* sp., *Hyalocladosporiella* sp., *Septobasidiaceae* sp., *Hortaea* sp., *Paraconiothyrium* sp., *Preussia* sp., *Sphaceloma* sp., *Trichomerium* sp., and *Rhytidhysteron* sp.) were identified in the mainland network. Connectors (*Myrothecium* sp., *Eriosporella* sp., *Trimorphomyces* sp., *Capnodiales* sp., *Fungi* sp., *Curvularia* sp., *Corticiaceae* sp., and *Glomerella* sp.) were also found in the island network, but no module hubs were detected. Members of Dothideomycetes were the most prominent keystone taxa (50%), followed by Sordariomycetes (25%). A large proportion of keystone OTUs (62.5%) had low relative abundance (<0.05%).

## Discussion

### Taxon identification

The phyllosphere fungal microbiome of *M. kwangtungensis* was largely comprised of Ascomycota taxa and dominated by members of Dothideomycetes and Eurotiomycetes at the class level (Fig. 2C). This outcome concurs with previous researches on the phyllosphere fungi in *Sequoia sempervirens*^83^, *Picea glauca*^17^, and *Mussaenda pubescens* var. *alba*^16^ using high-throughput sequencing methods. Kembel and Mueller^14^ found that the phyllosphere Dothideomycetes and Eurotiomycetes members across 51 tree species in a Panamanian rainforest contained a large number of melanized black yeasts, which were usually isolated from stressful environments and were remarkable for the wide variety of extremes they can tolerate. The most abundant genus in our study (*Aureobasidium*) is also known as a group of cosmopolitan black yeasts due to its melanin production^84^, which can help it withstand high ultraviolet (UV) radiation and moderately osmotic condition^85^. This stress-tolerance may be an important adaptation for the survival of fungi in a phyllosphere environment with low nutrient availability, strong UV exposure, and high temperature fluctuations throughout the diurnal rhythm^14,86^. Additionally, *Aureobasidium* spp. exhibit antagonistic activity against a number of phytopathogens, thus protecting host plants from disease^87^. Other high frequent Ascomycota species, such as *Cladosporium* spp. and *Phyllosticta* spp., may also offer enhanced stress tolerance for the host plant and have been frequently isolated from the leaf endosphere in previous studies^88–91^. Basidiomycota was the second most abundant phylum with Tremellomycetes as its dominant class. This class has previously been reported in foliar fungal communities associated with vascular plants in Svalbard, Norway^92^. A high abundance of Basidiomycota yeasts (*Papiliotrema*, *Symmetrospora*, and *Cryptococcus*) were detected in the phyllosphere fungal community of *M. kwangtungensis*. A similar scenario was also observed in the cereal phyllosphere mycobiome^93^. Andrews and Harris^94^ suggested that yeasts appear to be more active and permanent colonizers of leaf surfaces, whereas the filamentous fungi were mostly transients and often existed on the leaves as dormant spores.

### Community differentiation

The ANOSIM results suggested that phyllosphere fungal community structure differed significantly between mainland and insular habitats, even resembling the patterns in AMF community assemblages found by da Silva *et al*.^39^. This structure pattern was mainly influenced by host plant genetic structure (Table 1, Fig. 3). Plant genetic structure results from the joint action of migration, mutation, selection and drift^95^, thus reflecting the historical and biological context of plant species and determining their evolutionary potential^96^. One of the reasons for this significant population structure for *M. kwangtungensis* may be the relatively limited gene exchange due to the oceanic barrier. In addition, small and spatially isolated populations will lead to allelic fixation at some neutral loci within populations and subsequently promote population genetic divergence, mainly as a result of stochastic events or genetic drift^95,96^. We found lower genetic diversity displayed in island populations, which corroborate the traditional idea^35^. Founder effects, small population size, genetic bottlenecks, and geographical isolation from the mainland were commonly considered as major factors responsible for such a pattern arising^36^.

Host genetic divergence may result in variation in phenotypic traits including leaf chemistry, morphology or physiology, even within a single plant species^97–99^. The assembly and development of phyllosphere fungal communitis must involve those plant phenotypic traits controlled primarily by their genetic make-up^6^. For example, Saunders and Kohn^100^ demonstrated that benzoxazinoid production by host plant improved the ecological success of *Fusarium* species in the host. In addition, non-*Fusarium* species with intermediate 2-benzoxazolinone tolerance levels showed a colonization advantage over sensitive fungi, which indicates that phyllosphere fungal assemblage are at least partially filtered by host defense compounds. This conclusion was further confirmed through leaf-extract assays by Lau *et al*.^101^, who showed a strong effect of host versus non-host leaf chemistry on fungal endophyte growth *in vitro*. Additionally, leaf topographic features, such as trichomes, wax crystals and cuticle thickness, with variation occurring among intraspecies and cultivars, will also affect which and how many fungal species can persist in a location. For instance, trichomes or hairy extensions may ensnare water and fungal spores, or they may keep spores from adhering to the leaf surface^102^. Bailey *et al*.^103^ found that the establishment of some endophytic *Trichoderma* species were closely associated with glandular trichomes of *Theobroma cacao*. Moreover, leaf surface secretions (e.g., organic acids, simple sugars, and other easily utilized carbon source) have remarkably influenced the community structure of many yeast epiphytes^104^. Plant intraspecific phenotypic plasticity is now generally considered to be heritable and of potential importance for species’ evolution^97^. Therefore, adaptive plant genetic divergence among populations, which can result in local adaptation, may be the starting point for coevolution between a plant and its associated microbiome^105^. Balint *et al*.^106^ demonstrated that host genotype-specific phyllosphere fungal communities could live in the plant systemically, existing in the host tree even after two round of reproduction. We found strong congruence between host genetic divergence and phyllosphere fungal compositional divergence (Fig. 3), indicating an eco-evolutionary pattern in which evolutionary changes in the *M. kwangtungensis* associate with ecological changes in the fungal microbiome^107^. However, the functional basis and filtering mechanism for this obvious plant-fungi interaction still require further investigation.

MAP has a small but significant influence on structuring phyllosphere fungal communities (Table 1), which is in line with the patterns observed in foliar fungal communities of *Metrosideros polymorpha* across a Hawaiian landscape^13^, in AMF community assembly associated with four plant species in a grassland ecosystem^108^, and in endophytic fungal community of *Panicum hallii* across the Edwards Plateau^109^. Precipitation can directly influence the fungal species pool, and can also exert important influence on ecosystem processes including respiration, decomposition and plant productivity, as well as may indirectly affect plant-associated fungal communities via changes in the local host community properties^110^. Moreover, variation in environmental factors could also augment, weaken or veil the effects of host genes, which would lead to context-dependent expressions of genetic variation for plant phenotypic traits that may finally affect the assembly of phyllosphere fungal communities^111^.

### Co-occurrence networks

Investigating co-occurrence patterns among fungal community members can aid in detecting potential biological interactions, habitat affinities, or shared physiologies, and may provide new insight into the structure of complex fungal communities^112^. The potential biological interactions revealed by the ecological microbial network often range from positive (e.g. commensalism and mutualisms) to negative (e.g. predation and competition) relationships^113^. For example, fungal propagules may stimulate or antagonize growth of other phyllosphere members by producing metabolites, offering structural complexity, competing for resources, and changing foliar trophic webs^114^. In this study, the correlations among fungal OTUs in each network were revealed to be predominantly positive (Fig. 5), thus indicating the potential for widespread cooperative and syntrophic relationships between most fungal members, or multiple trophic levels, as well as the potential for sharing niches based on nutritional preference and functional distinctiveness in the phyllosphere micro-environment. This overwhelmingly positive association has also been reported in microbial networks associated with mosses, lichens and the bark of maple trees^29^, and in the rhizosphere zone of wild oat^115^.

Comparison of the phyllosphere fungal co-occurrence networks revealed significant structural differences between island and mainland populations (Fig. 5). The island fungal subnetworks were significantly less complex, less densely interconnected and more compartmentalized and modular than their mainland counterparts. The most important explanation for the topological differentiation is the genetic background of the host plant (Table 2). Host genotype could act as an important plant-imposed filter by preventing the establishment of species that lack the phenotype required to survive, and thus further influencing the fungal distribution and interactions. In addition to direct interspecific microbial interactions, interactions could also be indirect, via their effects on plant defenses and the chemistry of leaves^116,117^. For example, a colonizing fungal species may induce a host immune defense reaction, triggered by well-conserved traits (e.g., chitin) that are produced by current fungal inhabitants^98,118,119^. We also identified a significant effect of MAP on topological variations across the regions. This indicates that water-energy dynamics may play a critical role in determining co-occurrence patterns at a regional scale. Precipitation variability could rapidly alter key carbon cycling processes of plants (such as net photosynthesis), which would result in long-term consequences for carbon storage and biotic-atmospheric feedbacks^120^. Further, it has been reported that topological features of fungal co-occurrence networks are closely related to efficient carbon utilization^121^. Therefore, more connected networks are expected to have higher carbon uptake, as in the mainland.

Keystone OTUs are considered to have potentially crucial roles in network structure maintenance and may exert a large influence on community assembly^77,122^. The disappearance of these keystone nodes may segregate networks into more modules. The island network has fewer keystone taxa, which could partly contribute to its more modular and compartmentalized network structure. Keystone nodes may also possess vital ecological functions in the phyllopshere fungal community. Some connectors and module hubs detected in our study, such as *Acremonium* sp., *Preussia* sp., *Curvularia* sp., *Phomopsis* sp., *Rhytidhysteron* sp., *Fusidium* sp., *Glomerella* sp. and *Phoma* sp., have been frequently identified in previous studies as foliar fungal endophytes^123–129^. They are widely acknowledged as important determinants of host-plant fitness, productivity, and function. We found that more than half of the keystone nodes had relatively low abundances, as has been previously reported in soil microbial networks^77^. This suggests that rare microorganisms can also play functionally important roles in maintaining microbial networks. Future research focusing on uncultured keystone fungal species will be critical to understanding the mechanisms in network assembly better.

In conclusion, the phyllosphere fungal community structure and co-occurrence patterns of *M. kwangtungensis* showed significant dissimilarity between mainland and island populations. This differentiation was determined first by divergence in host plant genetics and secondarily by environmental variation. This study contributes to a richer understanding of the interplay between host plants and phyllosphere fungal microbiomes. However, much more research is needed to further explore how host plants influence niche differentiation and interspecific interactions in phyllosphere fungal communities, as well as how these influences may shape community structural variability, co-occurrence patterns and also long-term eco-evolutionary processes of plant-fungi interactions.

## Supplementary information

Supplementary information
