# Supplementary material for: Mainland and island populations of Mussaenda kwangtungensis differ in their phyllosphere fungal community composition and network structure
Source: Sci Rep. 2020 Jan 22;10:952. doi: 10.1038/s41598-020-57622-6 (PMC6976661; doi:10.1038/s41598-020-57622-6)
Supplement: Supplementary file 1 — Supplementary information [file 41598_2020_57622_MOESM1_ESM.pdf]

Supplementary materials

Mainland and island populations of *Mussaenda kwangtungensis* differ in their phyllosphere fungal community composition and network structure

Xin Qian[1,2], Shengchun Li[2], Binwei Wu[3], Yonglong Wang[3], Niuniu Ji[3], Hui Yao[3], Hongyue Cai[2], Miaomiao Shi[2,4]*, Dianxiang Zhang[2]*

[1]College of Life Science, Fujian Agriculture and Forestry University, Fuzhou, 350002, China

[2]Key Laboratory of Plant Resources Conservation and Sustainable Utilization, South China Botanical Garden, Chinese Academy of Sciences, Guangzhou 510650, China

[3]University of Chinese Academy of Sciences, Beijing 100049, China

[4]Center of Conservation Biology, Core Botanical Gardens, Chinese Academy of Sciences, Guangzhou 510650, China

*Authors for correspondence:

Dianxiang Zhang: dx-zhang@scbg.ac.cn; Tel : +86 (20)37252543

Miaomiao Shi: mmshi@scbg.ac.cn; Tel : +86 (20)37252543

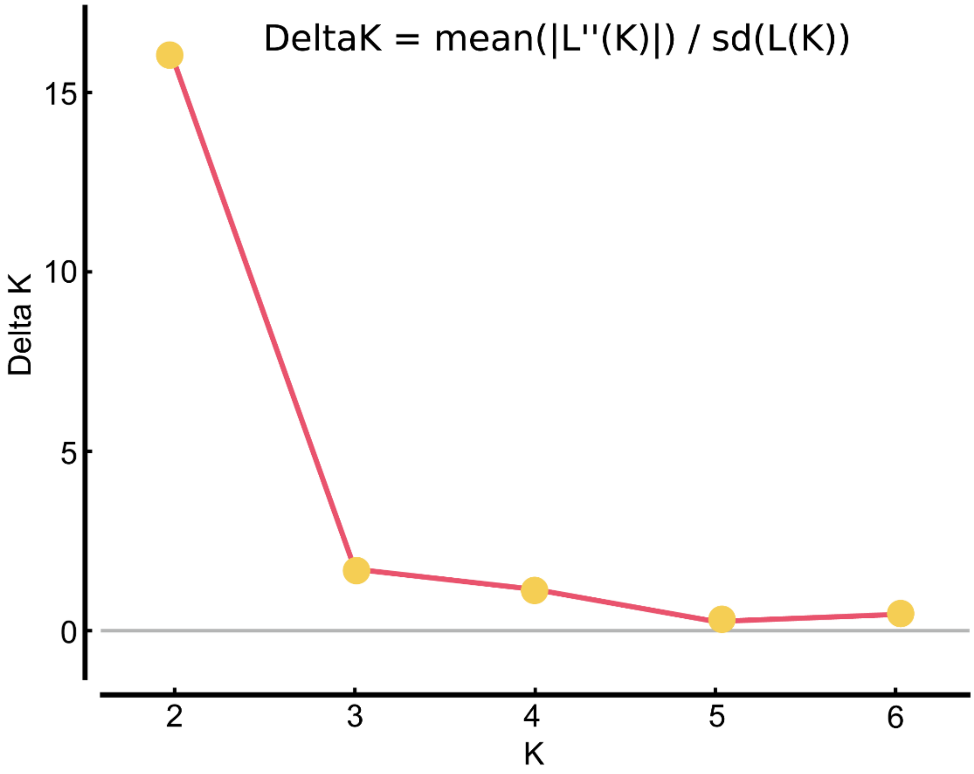

Fig S1. Best K as determined by calculating Delta K using STRUCTURE HARVESTER analysis.

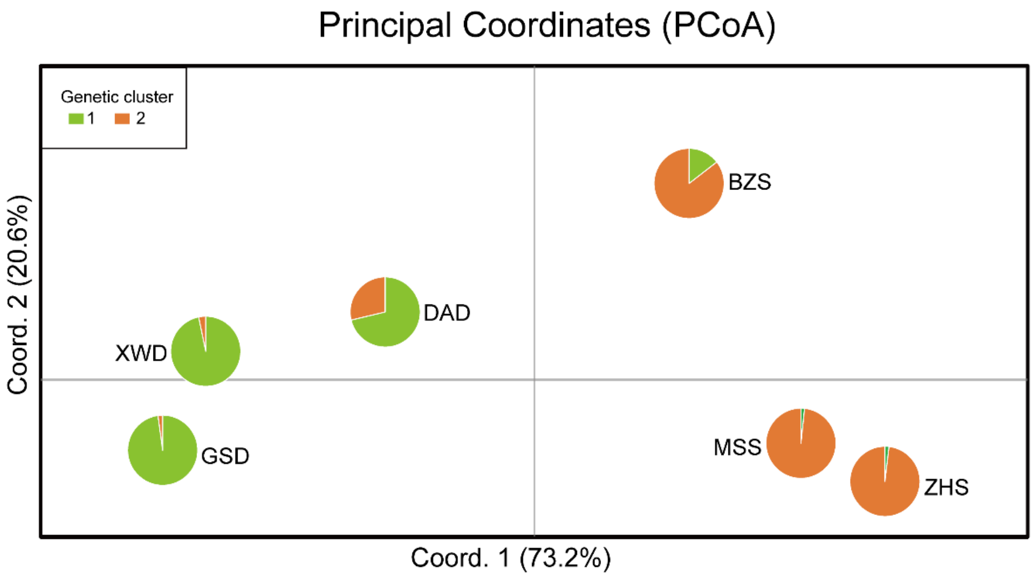

Fig S2. Principal coordinates analysis of variance based on DNA microsatellite data. Principal coordinate 1 and 2 account for 73.2% and 20.6% of the variation, respectively. Genetic clusters are identified by color in the pie chart.

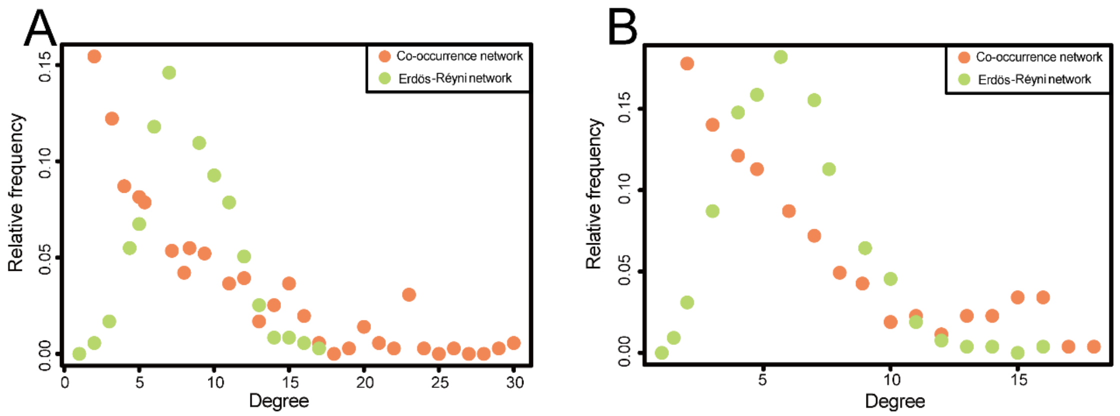

Fig S3. The distribution of degree for co-occurrence networks and Erdös-Rényi networks in mainland and island habitats.

Table S1 Population details of *Mussaenda kwangtungensis*

Population code	Region	Latitude (N)	Longitude (E)	Elevation (m)	MAT (°C)	MAP (mm)	*Ho*
BZS	Mainland	22.25	113.55	97	22.45	1983	0.600
ZHS	Mainland	22.21	113.48	76	22.64	2077	0.654
MSS	Mainland	22.35	113.57	51	22.53	1933	0.572
DAD	Island	22.02	113.71	70	22.40	1849	0.530
GSD	Island	22.13	113.82	35	22.41	1716	0.418
XWD	Island	21.95	113.69	65	22.44	1879	0.600

MAT: mean annual temperature; MAP: mean annual precipitation; *Ho*: observed heterozygosity

Table S2 Information of 11 SSR primers applied in the population genetic studies in *Mussaenda kwangtungensis*.

Primer	Sequence (5'-3')	Repeat	Size (bp)	T_m (℃)
AC30	F: GAAAATCCAAGAAACACAT	$(TG)_5TT(TG)_4$	435–463	Touchdown
	R: GACAACTCACAAGCCACTC			
CAA92	F: GGAAAAGATGACGGTTTGG	$(AG)_5AC(AG)_4$	194-216	58
	R: TAGTGATAAGCACGCCTGG			
CT12	F: CAAACTCGCTTCAAAAAAGTGACCATT	$(CT)_{10}$	223–249	56
	R: CAAACTCGCTTCAAAAAAGTGACCAT			
CT17	F: CCACAAAAAAGTAAACGCATA	$(TC)_6TT(TC)_4$	285–323	56
	R: CTCCCCTCTCACTGTAGAGAG			
CT48	F: CGGTAAAAAAAAGGATGGAGA	$(CT)_{19}$	316–350	53
	R: ATGGTATTGCGAGATGGAAAA			
CT59	F: ATTCCAGACACTTACTCACAGC	$(CT)_{11}$	251–299	56
	R: TGCAAACATACTTGATCCTACC			
CT60	F: CCTATATACTTGGTCTTGTGGT	$(TC)_{10}$	204–276	58
	R: CAGAACTATCTTATCTGTTGCC			
CT99	F: CGGGGAGGTCTTGAAAGA	$(CT)_7$	194–208	52
	R: AGAGGAGGAGGTGGCGAT			
CT113	F: AACATACAGACCCAAGCC	$(GA)_9$	276-328	Touchdown
	R: AAGCACCTACGAACTCCC			
CT135	F: CAAAGCAAAGGATAGTAGGA	$(AG)_{22}$	181–257	Touchdown
	R: GTTGACAGATGCTGGTAATG			
CT142	F: CACTGGAGAAGAAAAGCG	$(CT)_{17}$	255–311	Touchdown
	R: GCATGTGCATATACCCGA			

T_m: annealing temperature.
